# A multi-criteria decision analysis approach to assessing malaria risk in northern South America

**DOI:** 10.1186/s12889-016-2902-7

**Published:** 2016-03-03

**Authors:** Temitope O. Alimi, Douglas O. Fuller, Socrates V. Herrera, Myriam Arevalo-Herrera, Martha L. Quinones, Justin B. Stoler, John C. Beier

**Affiliations:** Abess Center for Ecosystem Science and Policy, University of Miami, Coral Gables, FL USA; Department of Geography and Regional Studies, University of Miami, Coral Gables, FL USA; Centro de Investigación Científica Caucaseco, Cali, Colombia; Faculty of Health, Universidad del Valle, Cali, Colombia; Department of Public Health, Universidad Nacional de Colombia, Bogota, Colombia; Department of Public Health Sciences, Miller School of Medicine, University of Miami, Miami, FL USA

**Keywords:** Malaria, Malaria risk, Multi-criteria decision analysis, Risk maps, South America

## Abstract

**Background:**

Malaria control in South America has vastly improved in the past decade, leading to a decrease in the malaria burden. Despite the progress, large parts of the continent continue to be at risk of malaria transmission, especially in northern South America. The objectives of this study were to assess the risk of malaria transmission and vector exposure in northern South America using multi-criteria decision analysis.

**Methods:**

The risk of malaria transmission and vector exposure in northern South America was assessed using multi-criteria decision analysis, in which expert opinions were taken on the key environmental and population risk factors.

**Results:**

Results from our risk maps indicated areas of moderate-to-high risk along rivers in the Amazon basin, along the coasts of the Guianas, the Pacific coast of Colombia and northern Colombia, in parts of Peru and Bolivia and within the Brazilian Amazon. When validated with occurrence records for malaria, *An. darlingi*, *An. albimanus* and *An. nuneztovari s.l.*, *t-*test results indicated that risk scores at occurrence locations were significantly higher (*p* < 0.0001) than a control group of geographically random points.

**Conclusion:**

In this study, we produced risk maps based on expert opinion on the spatial representation of risk of potential vector exposure and malaria transmission. The findings provide information to the public health decision maker/policy makers to give additional attention to the spatial planning of effective vector control measures. Therefore, as the region tackles the challenge of malaria elimination, prioritizing areas for interventions by using spatially accurate, high-resolution (1 km or less) risk maps may guide targeted control and help reduce the disease burden in the region.

## Background

Malaria continues to exact a toll in many developing countries where it is endemic through the economic and health burden it imposes. Malaria has historically contributed to increased health costs, decreased productivity, and slow rates of economic growth in 80 developing countries [1]. An estimation in 2013 showed that about 198 million cases and 584,000 deaths related to malaria occurred globally [2]. Although sub-Saharan Africa bears a disproportionately larger burden of the disease, South America also bears a significant case burden, with approximately 427,000 confirmed cases and 82 deaths in 2013 [2]. Of these, the nine countries in northern South America (NSA) accounted for ~ 90 % of the malaria cases in the continent [3]. Despite these figures, there have been vast improvements in malaria control in the past decade [2], so much so that malaria elimination in the NSA now seems feasible in the foreseeable future.

Global efforts to eliminate malaria such as the Roll Back Malaria program aim to “shrink the malaria map by progressively eliminating malaria from endemic margins inward” [4]. Achieving malaria elimination in the NSA, as in other region, will involve the systematic and synergistic use of multiple strategies including targeting areas for malaria interventions based on a stratification of risk. Spatially accurate, high-resolution risk maps delimiting areas of likely human-vector contact would not only help prioritize areas for malaria intervention, but also aid monitoring and evaluation of such interventions [5].

The stratification of risk depends on how risk is defined, yet there is currently no standard definition. Risk definitions have been dependent on the subject matter or purpose of the investigation [6]. Risk is broadly defined in public health as “the probability of disease developing in an individual in a specified time interval” [7]. Malaria risk is however not clearly defined due to the complexity of the disease that involves multiple hosts, vectors, and pathogens. Malaria risk has been defined using human cases (e.g. incidence and prevalence [8]), probability of *Plasmodium* presence [9], intensity of transmission [10], or its vectors (e.g. vector exposure [5], vector presence [11], and habitat suitability of vectors [12]). Thus, malaria risk is broadly considered as an array of factors that relate to the presence and density of vectors and parasites, all of which vary in space and time.

The direct estimation of malaria risk often involves malaria diagnosis and its relationship to populations at risk [13], but periodic, field-based survey data are typically limited in space and time in developing countries. Alternatively, in areas with limited data, malaria risk may be estimated indirectly through environmental covariates, which often show strong associations with malaria and mosquito distributions. The combination of these environmental surrogates in geographic information system (GIS) decision-support algorithms can reveal unexpected spatial patterns of malaria risk at unprecedented spatial resolutions [5]. Many types of spatial data derived from remotely sensed observations such as digital elevation models from the Shuttle Radar Topography Mission (SRTM) are now publicly available for most parts of the world, thus facilitating the potential estimation of malaria risk across large areas across multiple political units [5].

One method of mapping disease risk with limited field-based epidemiological or vector data is multi-criteria decision analysis (MCDA). This approach is preferred for its participatory framework, which employs statistical methods and human intuition, allows expert interaction, and accommodates non-linear relationships common between disease organisms and the environment [14, 15]. MCDA allows the combination of multiple environmental factors in estimating disease risk by employing decision rules derived from existing knowledge or hypothesized understanding of the causal relationships leading to disease occurrence [5, 15]. The output is a composite map which indicates lower or higher potential of disease occurrence in a location relative to surrounding areas on the same map [16]. MCDA has been useful in assessing risk of vector-borne diseases such as predicting suitable areas for rift valley fever in Africa [17], prioritizing areas of tsetse fly control in Zambia [18], malaria vector control in Madagascar [19] and risk of malaria vector exposure in parts of South America [5]. Building on the work by Fuller et al. [5], we set out in this study to evaluate malaria risk in the NSA based on environmental factors to produce risk maps that could guide targeted malaria interventions and potentially accelerate the drive towards malaria elimination in the region.

## Methods

### Study area

**Fig. 1:**
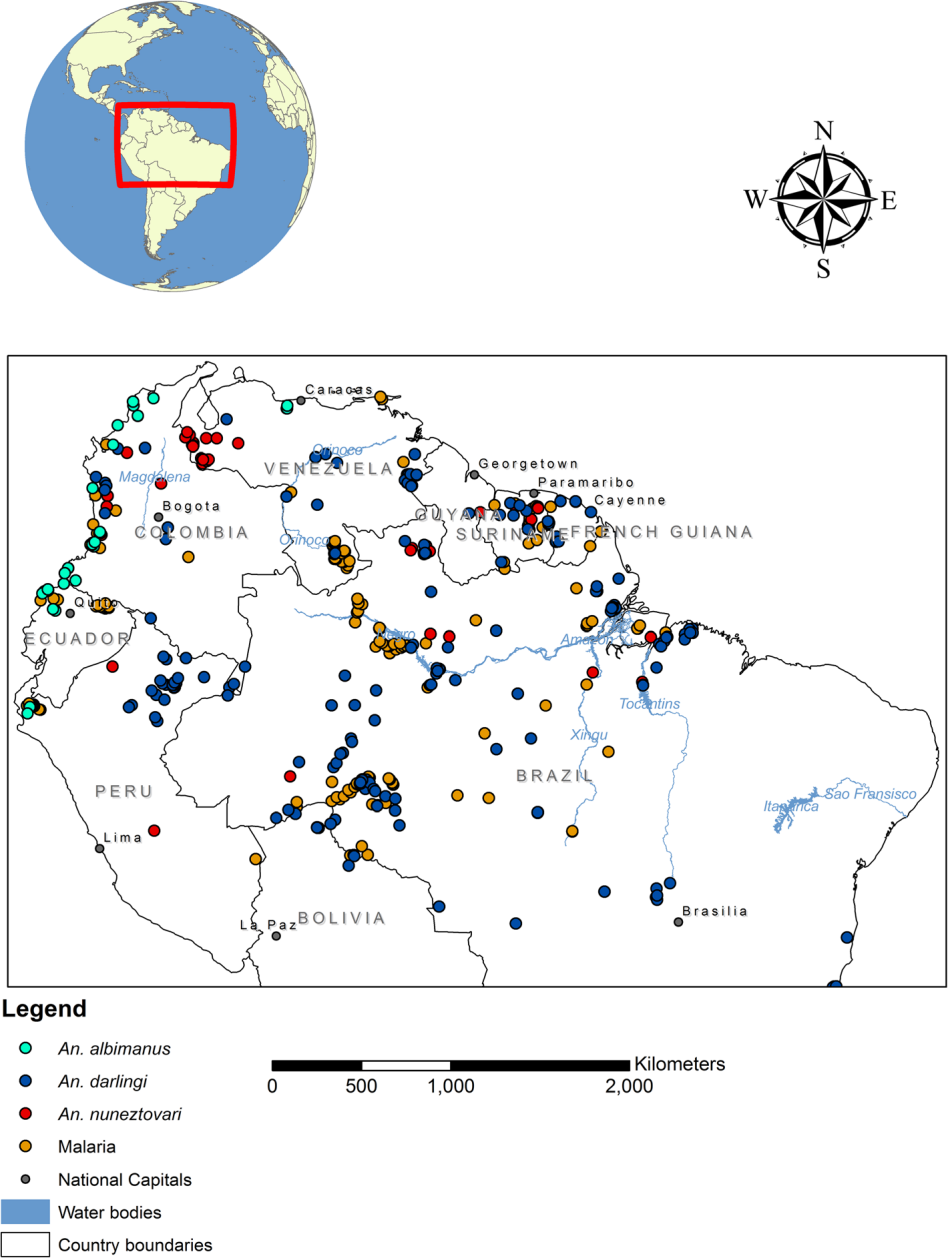
Map of the NSA showing *An. albimanus*, *An. darlingi*, *An. nuneztovari* s.l. and malaria sample locations

The NSA comprises of Colombia, Ecuador, French Guiana, Guyana, Peru, Suriname, Venezuela, and parts of Bolivia and Brazil (Fig. 1). The climate of the NSA is predominantly tropical, i.e., hot, wet and humid, especially within the Amazon rainforest and along the Atlantic and Pacific coasts [20]. Areas in the East around the Andes have high elevations (average height of 4, 000 m) and cooler weather (mean temperature range 18–22 °C) [21]. The vegetation follows a similar pattern: the tropical rainy regions and the Amazon basin have dense rainforests, while the savannas dominate in areas of highly seasonal rainfall such as the Llanos of Colombia and Venezuela and parts of central Brazil [22]. Vegetation along the humid slopes of the Andes also vary as elevation increases, with tropical trees at lower altitudes giving way to sub-tropical trees and finally grasses at higher elevations [22]. The countries also exhibit socio-economic differences which affect land use patterns and invariably disease incidence. For example, the large-scale soybean production in Brazil has not only led to increased revenue from external trade, but also higher deforestation [23], which has been linked to increased malaria and mosquitoes [24, 25].

Amongst the reported malaria infections in the NSA, *Plasmodium vivax* accounted for 75 %, followed by *P. falciparum* (25 %) [2]. Although malaria control is firmly established in the nine countries and reported cases are declining, only Ecuador is currently in the pre-elimination phase [2]. Many *Anopheles* vectors have been implicated in malaria transmission in the region [11, 26], and *An. albimanus* (Wiedemann 1820), *An. darlingi* (Root 1926) and *An. nuneztovari s.l.* (Gabaldon 1940) are the dominant species. All three vectors can transmit both *P. falciparum* and *P. vivax* [25, 27] and are anthropophilic [28, 29]. They are night biters exhibiting exophagic and exophilic feeding [27, 30–35]. They have been found in a wide range in the study area, including Colombia [34, 36], Amazonian plains of Ecuador [37], the Amazonian South and western Venezuela [38] and Brazil [39].

### Data sources

Sample locations for both parasite species were obtained through the Malaria Atlas Project (MAP) website. The data comprises surveys conducted by researchers and organizations between 1985 and 2009 in the various countries. Downloaded data also contained geo-referenced location of cases, the diagnostic method used for detection, age, the number of individuals examined, and number of individuals with parasites in the blood. Similar georeferenced data (Fig. 1) for the 3 vector species were obtained through the Walter Reed Biosystematic Unit [40] and the Global Biodiversity Information Facility [41]. These records included locations where both larvae and adult *An. darlingi*, *An. albimanus* and *An. nuneztovari s.l* had been sampled by different investigators between 1980 and 2007.

### Variable selections

Nine parameters associated with the environment, including climate were chosen based on their association malaria and its vectors (Table 1). These included factors related to availability of vector breeding sites (wetlands, precipitation and topographic wetness index -TWI, which was derived from the digital elevation model), thermal and altitudinal limits for parasites and vectors (elevation and temperature), and access to blood meals (population density, roads, urban areas and deforestation).

**Table 1:** Risk factors and fuzzy membership functions used to create risk maps

Data	Source	Factor	Control points	Fuzzy function	Rationale
Deforestation	Global Forest change [54]	Distance (km)	0, 5	Linear ↓	Vectors are found within 5 km of deforested areas
Elevation	SRTM 90 m	Elevation (m)	500, 1800	J-shaped ↓	Exposure to vectors decrease above 500 m and is non-existent above 1800 m
Population	LandScan	Population density	2, 50, 100, 150	Sigmoidal ↑↓	Populations between 2 and 150/km^2^ are sufficient for malaria transmission
Precipitation	WorldClim	Precipitation (mm)	0, 80	Linear ↑	Precipitation of 80 mm is suitable for vectors for stable transmission to occur [43]
Roads	DCW	Distance (km)	0, 5	Linear ↓	Transmission occurs within 5 km of roads where blood meals are available
Temperature	WorldClim	Temperature °C	18, 22, 32, 40	Sigmoidal ↑↓	Sporogony starts at 18 °C and is completed at 22 °C, vector survival decreases above 32 °C and death occurs at 40 °C [43]
TWI	SRTM 90 m	Soil Saturation (%)	0, 5	Linear ↑	An area requires about 5 % water saturation to serve as breeding site
Urban areas	DeLorme, Inc.	Distance (km)	1, 10, 20, 30	Sigmoidal ↑↓	Vectors are absent in urban areas but found in the urban periphery
Wetlands	WWF	Distance (km)	0, 3	Linear ↓	Vectors are found within 3 km of wetlands

*Abbreviations and Symbols*: *SRTM* Shuttle Radar Topography Mission, *DCW* Digital Chart of the World, *WWF* World Wildlife Fund. The ↑ arrows indicates an increasing function, ↓ a decreasing function and ↑↓ a symmetric function

### Procedure

#### Risk map generation

Two data layers (elevation and TWI) were resampled to 1 km spatial resolution to maintain consistency with the other layers originally provided at 1 km. Resampling was carried out using the nearest neighbor algorithm, which preserves original data values. A binary discrete raster was created from the elevation layer to serve as a constraint, excluding areas with elevation >1800 m where risk of transmission was assumed to be negligible [5]. Because the influence of categorical variables on risk of malaria and vector exposure was based on access (Table 2), we created distance layers measuring proximity to the features before further analyses.

**Table 2:** Factor groupings and weights used for risk maps

Factor	Factor groupings	Factor weight			
		AHP^a^	Equal^b^	Access related^c^	Environment/Climate related^d^
Distance from deforested patches	Access	0.0996	~0.11	0.14	0.06
Population density		0.0593			
Distance from roads		0.0379			
Distance from urban areas		0.0420			
Distance from wetlands		0.1391			
Elevation	Environmental/Climatic	0.1680		0.075	0.175
Precipitation		0.1784			
Temperature		0.2006			
TWI		0.0751			

^a^Factors weighed based on ecological relationship with mosquitoes and malaria

^b^No difference in weighting

^c^Access more important (group weight sum up to 0.70)

^d^Environment/Climate related factors more important (group weight sum up to 0.70)

*TWI* Topographic Wetness Index

The data layers contained variably scaled information; hence, fuzzy functions were employed to standardize all the layers to a common data range needed to facilitate factor integration. Fuzzy functions measure the degree of membership of data cells in a layer through control points that are set based on the relationship between the layer and disease/vectors. These relationships determine the shape (linear, sigmoidal or J-shaped) and direction (increasing, decreasing or symmetric) of the fuzzy function (See Table 1), which were represented on an 8-bit (0–255) scale in our analysis. For instance, we used a linear decreasing function to scale risk associated with access to blood meals such as deforestation by assuming highest risk when close and no risk when more than 5 km away from the feature.

Prior to use in the MCDA, each fuzzy layer was assigned a weight indicating its importance in the risk assessment. To facilitate the process of weighting, the nine factors were combined into two logical groups: (i) access-related factors relying on distance/proximity to features; and (ii) environment/climate related factors (Table 2). Weights were subsequently assigned in four ways: (i) by weighing all factors equally; (ii) assigning higher weights to access-related variables; (iii) scaling environment/climate related factors higher (approximately three-quarters of total weights assigned to group of factors with higher weighting in each case); and (iv) assigning weights based on interaction between factors and disease/vectors using the analytical hierarchical process (AHP). The AHP assigns weights to each factor by assessing the relative importance of factor pairs in a pairwise matrix [42]. Pair comparisons were conducted by evaluating the importance of each factor relative to the other in a pair and assigning values ranging from 1 (extremely less important) to 9 (extremely more important). Evaluation for 6 of the factors were carried out by a group of malaria experts in a risk mapping workshop in Cali, Colombia (details of procedure published elsewhere [5]). Our ranking of the other 3 factors was based on literature searches by which we determined that temperature, precipitation, and deforestation be ranked in descending order [43]. The principal eigenvector was subsequently used to determine the final weight of each factor. The consistency of the pairwise matrix was evaluated using a threshold of 0.1, a ratio above which the pairwise matrix should be revised while values below indicate acceptable consistency [42]. Table 2 shows all factor weights assigned using the AHP and the other methods.

Finally, the multi-criteria evaluation (MCE) module was used to integrate all data layers to create composite risk maps for the study area. A number of user-specified options exist in the MCE module for this purpose but for our analysis, we chose the weighted linear combination (WLC). The WLC is a linear function which combines fuzzy layers according to their weight of importance (all factor weights add up to 1) [5, 44, 45], producing final composite maps of risk based on the four weighting methods. All analyses were conducted using the raster-based GIS software, Idrisi (Selva edition) [45].

#### Assessment of risk maps from sample points

Resulting risk maps were evaluated by comparing differences in mean risk scores between randomly generated points (*n* = 1502) and the risk scores at the sample locations of *An. darlingi* (*n* = 168), *An. albimanus* (*n* = 38), *An. nuneztovari* s.l. (*n* = 114) and malaria cases (*n* = 218) respectively. Assuming normal distribution, differences between the mean risk scores for each vector and malaria occurrence points and random control points were assessed using unpaired *t-*test. A one-way analysis of variance (ANOVA) was used to compare the means of the four groups of sample points. Both statistical analyses were performed in SPSS v. 21 software [46]. Spatial autocorrelation of the sample points was tested using the Moran’s *I* statistic in ArcGIS 10.2 software [47]. Moran’s *I* tests the null hypothesis that the attribute of the feature of interest is randomly distributed where a statistically significant *Z*-score indicates spatial autocorrelation. To correct autocorrelation found in sample points, we systematically excluded points until arriving at a distribution that was spatially independent.

## Results

### Malaria risk distribution

**Fig. 2:**
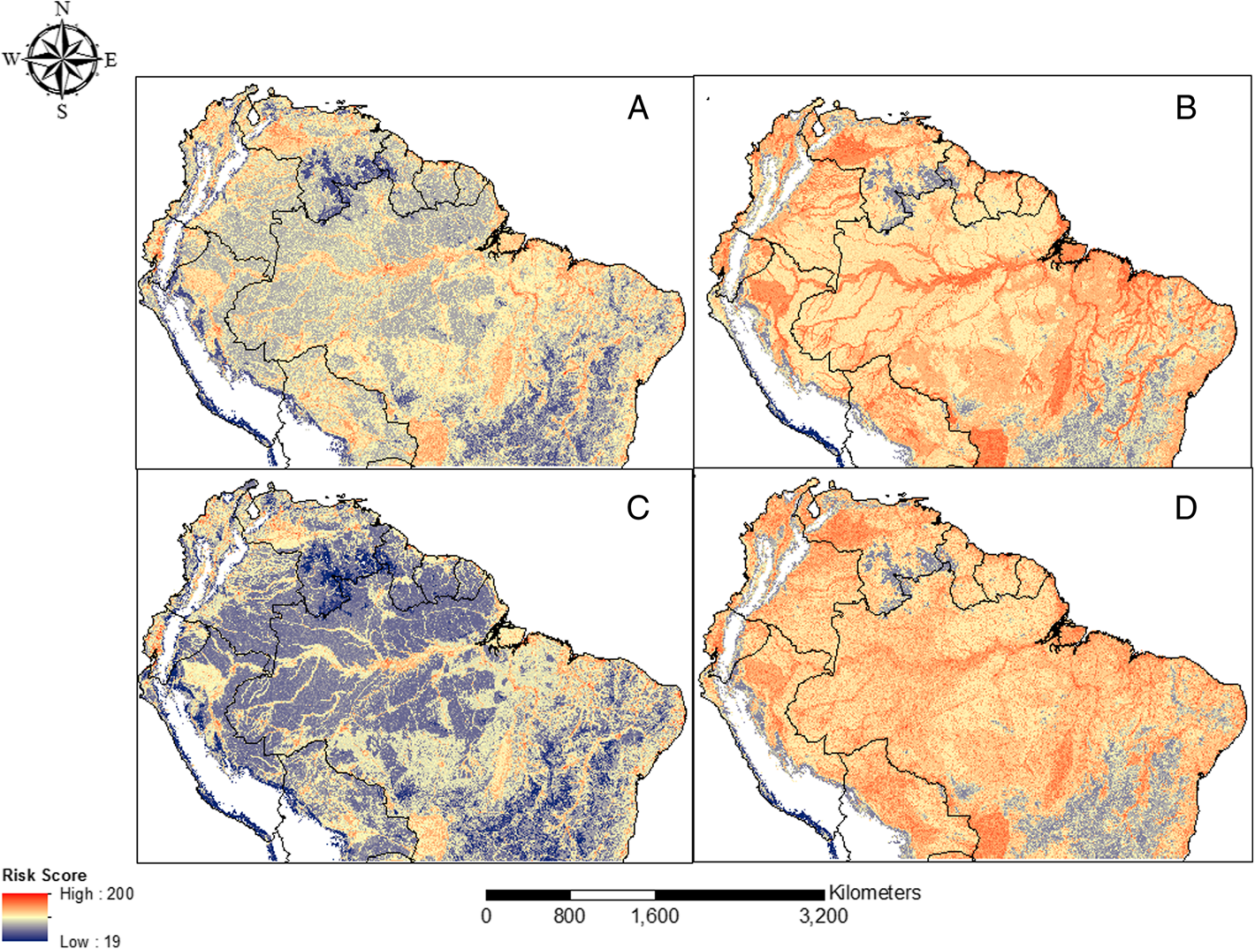
Risk maps derived from weighted linear combination of 9 factors. Higher values indicate relatively higher risk scaled from 0 to 255. **a** Each factor assigned an equal weight of 0.11; **b** Factors weighed according to ecological relationship with mosquitoes and malaria through AHP; **c** Access was assigned more weight (0.7 out of 1); **d** Environmental/Climatic factors was given more weight (0.7 out of 1)

The composite maps of risk produced using the four weighting methods are presented in Fig. 2a–d. In Fig. 2a, the risk map was produced by assigning an equal weight of 0.11 to each of the twelve factors. The composite layer in Fig. 2b included all the factors weighted through AHP. All five access-related factors in Fig. 2c were assigned equal weights, which summed up to 0.7, thus giving access-related factors a higher weighting than environment-related factors which had a total of 0.3. For Fig. 2d these weightings were reversed; the four environment-related factors were given a cumulative value of 0.7 while access-related factors were assigned a total of 0.3.

The different maps reveal noticeable differences in the level and distribution of risk. For instance, the distribution of risk in Fig. 2c is more heterogeneous compared to the other maps. In this composite layer, the Amazonian areas of Brazil, Venezuela, Colombia, the Guianas, and Peru, as well as southern Brazil and areas on the fringes of the Andes display low risk scores relative to areas outside the Amazon basin. The relatively higher weight given to access-related factors may account for this distribution particularly in the Amazon, as the area is associated with low population density and limited access via roads and rivers, hence the lower imputed risk. Areas of relatively moderate to high risk on this map were found mostly along stretches of rivers in the Amazon basin, along the coasts of the Guianas, in the seasonally flooded wetlands around the Llanos, in patches around south-western Brazil, in areas west of the Andes in Peru and Bolivia, along the coasts of Ecuador and Colombia, and in northern Colombia.

The areas delineated as moderate to high risk locations in Fig. 2c are common to all the maps; however, additional areas of high risk are highlighted in the other maps. Contrary to what was shown in Fig. 2c, the Amazon forest had elevated risk of transmission, particularly in the AHP guided map (Fig. 2b), which displays moderate risk relative to the other maps. In the equally weighted map (Fig. 2a), moderate to low risk can be seen especially throughout the Amazon basin, Southern Venezuela, and central Brazil. Although the AHP and the environment -related maps (Fig. 2b and d respectively) appear similar because the total weight assigned to environmental factors in both maps was similar (0.6221 and 0.7 respectively), differences in the maps are evident. High risk areas in Fig. 2b are displayed along the rivers in the Amazon basin, the wetlands, and along the coasts in the study area whereas risk is depicted in a spatially homogeneous fashion in Fig. 2d. Overall, similar areas of low risk are displayed in central Brazil, southern Venezuela and the Andean fringe while the high risk areas identified in all the maps are consistent with current understanding of malaria risk in the region [48, 49].

### Validation of risk maps from sample points

**Fig. 3:**
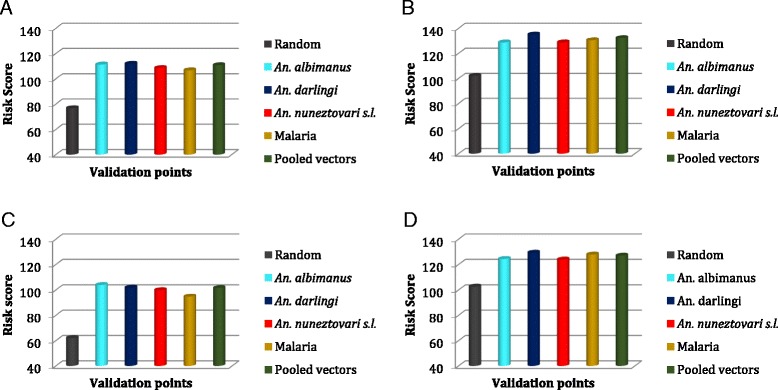
Mean risk scores for MCE models validated with vectors and malaria data points. **a** Equal weights for all 9 factors; **b** Factors weighed according to ecological relationship with mosquitoes and malaria using AHP; **c** Access factors have higher weighting; **d** Environmental/Climatic factors have higher weighting. Mean scores for all vectors and malaria points are statistically different from random at *p* < 0.0001

The test for spatial autocorrelation showed that vector occurrence points for *An. darlingi* (Moran’s I = 0.036, z = 0.07, *p* = 0.94) and *An. albimanus* (Moran’s I = 0.458, z = 0.68, *p* = 0.39) were spatially random within the study area. Autocorrelation was detected in *An. nuneztovari s.l* (Moran’s I = 0.758, z = 2.902, *p* = 0.03) and malaria (Moran’s I = 747, z = 8.632, *p* = 0.00) occurrence points. The z-scores however remained significant after systematically reducing the number of sample points (*n* = 90 and 172 for *An. nuneztovari s.l* and malaria respectively), thus suggesting that spatial dependence did not significantly influence results. Figure 3 shows the means from the MCE risk maps for the validation points. The *t-*test results indicated that mean cell-level risk scores at the occurrence locations were significantly different and higher (*p* < 0.0001) than risk scores of the random points (Table 3). Output from the one-way ANOVA test performed on 467 observations (Table 3: between and within group df) showed no significant difference in mean risk scores among occurrence points, suggesting that the occurrence points may be pooled into a single sample. Further analysis with *t*-test indicated that the pooled vector points were significantly different and higher (*p* < 0.0001) than randomly distributed points (Table 3).

**Table 3:** Validation of risk maps using t- test and One-way ANOVA

Models	Validation points						
	*t* statistic					ANOVA	
						Between groups (df)	3
						Within group (df)	464
	*An. albimanus* ^a^	*An. darlingi* ^a^	*An. nuneztovari* ^a^	Malaria^a^	Pooled vectors^a^	*F* statistic^b^	*p*-value
AHP	6.12	15.44	9.35	13.47	18.23	1.94	0.12
Equal	8.61	17.70	12.05	15.32	21.67	1.15	0.33
Access	9.77	18.57	13.33	15.49	23.06	1.84	0.14
Environment/Climatic	5.05	12.77	7.57	12.20	15.04	1.51	0.21

^a^Statistically different from random at *p* < 0.0001

^b^Comparison of means for *An. albimanus, An. darlingi, An. nuneztovari* and Malaria cases

## Discussion

### Spatial distribution of vector exposure and malaria risk

Using publicly available environmental, vector, and case data, our study elucidates the spatial distribution of malaria and potential vector exposure risk and provides important spatial information that may guide targeted malaria interventions in the region. Although the environmental parameters typically change very little or gradually over time [5], the inclusion of a deforestation measure reflects a highly dynamic landscape variable that is strongly associated with malaria risk. This is exemplified in our four composite maps, which show most areas in the Amazon and southern Brazil where deforestation has been most pronounced in the past decade [50] as having moderate-to-high risk of malaria.

Although there are common areas with moderate to high risk on all four maps, there are also areas of model over-estimation. While the risk surface in Fig. 2b aligns relatively well with known malaria risk [48, 49], the result of the access-related grouping is similar to that produced by Fuller et al. [5] for parts of the study area. Overall, based on Figs. 2 and 3, A and C provide a more realistic depiction of risk; however, it should be noted that malaria transmission does not occur along the Atlantic Coast of Brazil south of the Amazon basin; therefore, what the maps depict is more likely a better representation of risk of vector exposure than actually malaria transmission. Risk was however over-estimated in all four maps in areas around central and along the Atlantic Coast of Brazil south of the Amazon Basin where urbanization, transportation infrastructure, and environmental factors have favored vector control.

The consistently higher mean risk scores for *An. darlingi* and *An. albimanus* may also reflect their importance in malaria transmission in the region [25, 27, 51]. While *An. darlingi* is the predominant vector in the study area [27], *An. albimanus* is more wide spread particularly in Colombia and the northern-most portions of the study region [51].

### Comparison with previous studies

Further, whereas many previous risk-mapping exercises focus on individual political units, these maps show how risk is represented across political boundaries, whether national or local [48, 49]. While previous malaria risk maps show current risk based on actual malaria cases aggregated by municipalities [48, 49], our composite maps the effects of environmental and climatic conditions and their perceived degree of association with vectors and malaria transmission [5, 52]. Our approach avoids limitations of aggregating cases by municipality (e.g. giving no indication of the location of transmission or clustering of cases) by producing a continuous risk surface with high spatial detail and clearly defined risk gradients.

Unlike the weak relationship reported between malaria cases represented by municipalities and mean risk scores in Fuller et al. [5], mean risk scores for malaria points used in this study were consistently higher than at random locations. This may be the result of employing geo-referenced malaria point locations as this is more easily relatable to pixel-level risk scores than political units represented as polygons.

### Study limitations

The subjective nature of the MCDA approach in assigning fuzzy functions and weights undoubtedly produces some biased outcomes as well as probable inflation of risk scores when correlated variables are used [5, 15]. We also acknowledge the possibility of temporal and geographical bias in the sampling of occurrence points as a result of multiple collectors and the variable time of collection. Moreover, the dearth of up-to-date secondary and tertiary road network data for the study area may also have limited the estimation of risk based on access to roads, particularly in the northern parts of the study area.

## Conclusion

We evaluated the exposure of the NSA to malaria risk given current access-related and environmental/climatic conditions using MCDA. We produced high-resolution composite maps showing gradients of risk which were validated with geo-coded occurrence points for malaria and three dominant vector species. These new map products represent an improvement upon previously published map of malaria risk in the region, which was highly generalized and constrained by political boundaries [50, 53]. The incorporation of a deforestation layer representing land-use change, provided additional detail to the risk maps relative to past studies that have employed MCDA for malaria vector exposure risk [5]. This also revealed that our depiction of risk produced was related to malaria occurrence points. Despite limitations of the knowledge-based approach to risk mapping, our 1 km maps provide information to the public health decision makers/policy makers to give additional attention to the spatial planning of effective vector control measures. This may increase the potential for malaria elimination in the region in the near future.

### Ethics approval

Ethical clearance was not sought because human subjects were not involved.

## Abbreviations

AHP: analytical hierarchical process
ANOVA: analysis of variance
GIS: geographic information system
MAP: malaria atlas project
MCDA: multi-criteria decision analysis
MCE: multi-criteria evaluation
NSA: Northern South America
TWI: topographic wetness index
WLC: weighted linear combination
